# Dog Handler Beliefs regarding Barriers and Facilitators to Canine Health Promotion and Injury Prevention in Swedish Working Dog Trials and Competitions

**DOI:** 10.3390/vetsci9050242

**Published:** 2022-05-21

**Authors:** Ann Essner, Catarina Kjellerstedt, Amie L. Hesbach, Kristina Svensson, Helena Igelström

**Affiliations:** 1 IVC Evidensia Djurkliniken Gefle, Norra Gatan 1, SE-803 21 Gavle, Sweden; 2 Department of Women’s and Children’s Health, Uppsala University, SE-751 24 Uppsala, Sweden; helena.igelstrom@neuro.uu.se; 3 Veterinär Catarina Kjellerstedt, Östgötagatan 5, SE-186 35 Vallentuna, Sweden; catarinakjellerstedt@gmail.com; 4 EmpowerPhysio, 2585CW The Hague, The Netherlands; amiehesbach@gmail.com; 5 Tolleruds Gård 116, SE-655 91 Karlstad, Sweden; tollerudsgard@gmail.com

**Keywords:** qualitative research, content analysis, dog handler, sporting dog, working dog, animal welfare, canine health, well-being

## Abstract

Dog trials and competitions involve various sport disciplines, e.g., obedience, agility, working dog trials and rally obedience. Dog handlers navigate their dogs through physically and mentally demanding tasks. The purpose of this study was to gain a better understanding of barriers and facilitators to canine health promotion and injury prevention described by dog handlers. Methods: Qualitative inductive content analysis was applied to systematically organize and interpret narrative data from 654 respondents’ answers to open-ended questions in an anonymous online inquiry. Results: Two categories, with seven sub-categories, emerged from the analysis: (1) Challenges in applying the regulations in dog trials and competitions, and (2) Implementation of animal welfare and canine well-being approaches. Respondents described the challenges in applying regulations in dog trials and competitions and lack of scientific research as barriers to their intent to prevent injuries in their dogs. Implementation of animal welfare and canine well-being approaches were described as facilitators. Conclusion: The findings imply that the stakeholders continuously need to work on bridging possible gaps between the canine welfare criteria and the scientific and empirical knowledge in canine sports and performance medicine.

## 1. Introduction

Since 1918, working dog trials, obedience, rally obedience and agility competitions, dog and dog handler educational programs, mental tests, and structural and conformational evaluations have been organized by the not-for-profit Swedish Working Dog Association (SWDA) [1,2]. The SWDA is an association organized under the Fédération Cynologic Internationale (FCI), which is the international governing body for canine sporting disciplines. In addition, SWDA has the responsibility for setting breeding and health standards in approximately 25 working dog breeds in FCI breed groups 1 and 2, e.g., Rottweiler, Dobermann, German Shepherd, and Belgian Shepherd. Furthermore, the SWDA has a vital societal function in certifying military dogs for the Swedish military defence, search and rescue dogs for the Swedish Civil Contingencies Agency and the Swedish Maritime Administration, and assistance dogs as civil working dogs [3,4,5,6,7,8]. Assistance dogs are dog guides for the blind, hearing dogs, mobility assistance dogs, seizure and diabetes dogs, and psychiatric service dogs [3,9]. Sporting and working dog trials and competitions organized by the SWDA involve several disciplines, i.e., obedience, agility, messenger, patrol, protection, search, tracking, mondioring and rally obedience. In 2018, 178,265 registrations took place at competitions and working dog trials under the auspices of the SWDA.

During trials and competitions, handlers navigate their dogs through physically and mentally demanding tasks [10]. In many of the disciplines the dogs go through courses with obstacles to overcome and objects to retrieve. The obstacles are hurdle jumps of pre-determined heights and the objects are of various pre-determined sizes and weights in relation to the dog’s height at the withers [11,12,13,14]. In the working disciplines, dogs perform scent tasks, e.g., tracking and searching for people [13]. The dogs’ success depends on their ability to jump, retrieve objects, perform scent tasks, e.g., tracking and search for people, to focus in precision movement during heelwork, i.e., the dog’s shoulder remaining level with the handler’s leg throughout various maneuvers, and to complete tasks without faults according to a judge and dog sport-specific regulations [15,16]. Several of the tasks involve unnatural body positions and powerful movements with rapid directional changes in order to succeed with fewer faults and/or the highest scores possible [17,18,19,20]. All disciplines consist of progressive levels, from beginner to higher competitive levels. Each level consists of increasingly challenging exercises, e.g., heavier dumb bells, recalls with quicker stops, higher obstacles, longer tracks, and larger search areas in heavier terrain [12,13].

Over the last decade, new research has reported on dogs’ physical adaptations to stress [21,22,23,24] and care of sport and working dogs with regards to physical performance [25,26,27]. Identification of sport-related risk factors with regards to canine health and well-being have gained more attention among handlers of working and sport dogs [28,29,30,31,32,33,34,35]. Information on dog handlers’ perceived barriers and facilitators to sustainable working and sport dog health and well-being is, however, very sparse or non-existing.

There is also a growing unease and concern about the risk of injury in working and sport dogs and concerns surrounding the health of canine athletes, which may result in ethical dilemmas for stakeholders, i.e., dog handlers, breeders and organizers of competitions. Epidemiological research on the prevalence of injuries among sporting and working dogs other than in agility is sparse. Based on previous research, the prevalence rate of sport-related injuries in agility dogs varies with populations. Prevalence rates between 8–42% have been found in dogs suffering at least one sport-related injury during their career [28,31,33,36,37]. Levy et al., (2009) found that 11% of injured agility dogs were forced to retire from sports. Within the canine sport and working dog field, there are differences with regard to culture and management strategies among dog handlers who are trying to prevent injuries and promote health in canine sports athletes [29,33,38]. The handlers’ drive for competition success may potentially reduce their standard of health and well-being for the dog, and at the same time, the dogs are their canine sporting partners [39].

Among breeders, dog trial and working performance credentials are used alongside health indicators, e.g., hip and elbow scores, behavior and personality assessments, and structural and conformational trials, as tools in the selection of breeding dogs [40]. Credentials based on the dogs’ working performance represent working ability, as in the relationship between structure and function, real-world coping strategies and cognition, and, therefore, additional components of the dogs’ physical and mental capacities [10,41,42,43,44]. However, it is also well-known that working performance is moderated by variables including the experience of the dog handler and associative learning principles based on reward and punishment typically used in dog training [45,46,47,48].

There is an identified lack of research in experienced dog handlers’ perceptions regarding canine health promotion and injury prevention in trials and competitions. Hence, the aim of this study is to gain a better understanding of the dog handlers’ perceptions of barriers and facilitators to canine health promotion and injury prevention within the scope of the SWDA and in selected canine sports and working dog disciplines.

## 2. Materials and Methods

### 2.1. Study Design

This research was part of a wider series of investigations from a cross-sectional anonymous online survey of dog handlers regarding physical activity, sport-specific training, and injuries in their sport and working dogs. To address the research aim of the present study, two open-ended questions were constructed within the survey to explore this emerging and under-researched topic in canine performance medicine. In the two questions, the respondents could elaborate on perceived facilitators and barriers to canine health promotion and injury prevention in trials and competitions associated with the SWDA as a stakeholder:

- How do you suggest that the SWDA can work to prevent injuries in sport and working dogs?
- What would you like the SWDA to consider for the future revision of the internal regulations of trials and competitions to promote increased health in sport and working dogs?

Since the questions were open-ended and prompted elaboration on suggestions on improved injury prevention, a qualitative method using inductive content analysis was applied to systematically organize the textual data. We focused on the manifest content rather than the latent content since text-based data tend to imply less context and conceptual richness [49].

### 2.2. Participant Recruitment and Data Collection

Data were collected via a retrospective anonymous survey distributed electronically. The survey was developed and distributed by means of an internet survey site (Google Forms, Google LLC, Mountain View, CA, USA) to facilitate the data collection. The survey was initiated on 1 February 2019 and remained open for two months until 3 April 2019. Recruitment strategies included adverts with the survey link at the internet sites of the SWDA (2 January 2019 and 25 March 2019) and the Swedish Kennel Club (27 March 2019), and through social media groups focused on agility, obedience, utility and working dog trials, as well as various relevant Facebook groups. In the introducing text, potential participants were informed about the inclusion criteria: handler of a dog born 2005 or later, participation in utility or working dog trials organized by the SWDA at any level, access to the internet, and willingness and ability to complete a survey in Swedish. Participation was initiated when an interested individual clicked an embedded hyperlink that directly accessed the appropriate survey. The respondent could fill in the questionnaire for several dogs.

### 2.3. Content Analysis

Content analysis was chosen for data management, as it is appropriate for textual data collected in open-ended questions where respondents have been allowed to elaborate and identify issues not captured in closed questions in surveys [49]. The content analysis had an inductive approach and followed a process as identified by Elo and Kyngäs [50] and Graneheim and Lundman [51,52], consisting of preparation, organization, and reporting. Since text-based data tend to be less complex and rich compared to interview-based data [49], the analysis focused on the manifest (explicit or obvious) content rather than the latent (oblique) content [51]. The analysis was performed in a step-by-step manner: (1) the text was read through several times by the coders; (2) meaning units were identified; (3) each meaning unit was condensed and labelled with a code, preserving the core meaning; (4) the codes were sorted and abstracted into subcategories, illustrating the manifest content of the data; (5) the subcategories identified from the analyses of the dog sport competitors’ responses were merged, based on common content, and abstracted into categories.

The open survey answers were compiled, analyzed and categorized by three authors (A.E., C.K. and H.I.). The organizing of data was undertaken using Windows Excel 365 (Microsoft Corporation, Redmond, WA, USA). Meaning units were coded, the codes were sub-categorized and relationships between sub-categories and categories were identified. Trustworthiness in the coding and internal reliability of this study was controlled for through data triangulation and consensus between three coders (A.E., C.K. and H.I.) [51]. Two other authors checked for consistency between the data and the categories (A.H. and K.S.). Results were reported descriptively in table form, all in accordance with the consolidated criteria for reporting qualitative research (COREQ) [53]. To illustrate the depth of the written answers, the average number of words in the answers are reported.

In qualitative research, patterns in the perceptions of all respondents are represented, hence expressions were not quantified. Although a data-driven, i.e., inductive, approach was used for the analysis, the categories identified and the interpretations made are shaped by the background and experience of the authors. Additionally, there is personal experience within the research topic in the group of authors; A.E. is a licensed human and animal physical therapist and a researcher with more than 25 years of clinical experience in canine performance and dog sports. C.K. has been a veterinary surgeon for more than 30 years and an active working dog handler. K.S. is a licensed human physical therapist and a judge within the SWDA, A.H. is a licensed human physical therapist and canine rehabilitation practitioner and therapist and, finally, H.I. is a licensed human physical therapist and a senior researcher with comprehensive training and experience in the methodology.

### 2.4. Ethical Considerations

Survey participation, i.e., responding to an anonymous online questionnaire, was completely voluntary after a general invitation on webpages of the SWDA, the Swedish Kennel Club, and social media. The respondents were informed and free to choose whether to participate in the study.

This research was conducted on data reported from handlers of sport dogs without subjecting the dogs to any kind of stress or suffering. All human respondents were debriefed in writing regarding the aim of the study and informed that by completing and submitting the survey, the respondents were providing their informed consent. No personal or sensitive data were collected from the respondents and all data were anonymous. Retrospective questions about the respondents’ sporting dogs were asked. The design of the two open-ended questions implied that the human respondents were not subjected to methods aiming to affect them physically or mentally. Respondents could possibly withdraw their responses by contacting the responsible researcher (A.E.) and share descriptive data regarding the dog, e.g., age, sexual status, breed, weight, sport disciplines. Otherwise, the responses could not be traced back to detect individual responses.

The study was conducted in accordance with the national animal and human ethical guidelines provided by the Swedish codes of statutes: SFS 2018:1192 [54,55]; SJVFS 2019:9 [56]; SFS 2003:460 [57], and were in line with the guidelines of the Declaration of Helsinki [58].

## 3. Results

**Figure 1:**
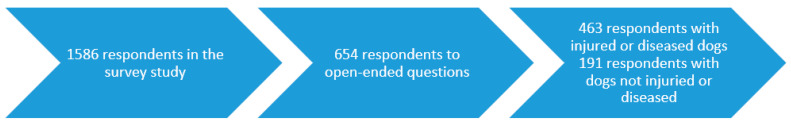
Flow chart illustrating enrolled respondents.

There were 1586 dogs included in the survey study. Six-hundred and fifty four respondents provided textual data in response to two open-ended questions. Among the respondents, there were 463 reporting injury or disease in their dog, whereas 191 reported no injury or disease (Figure 1). On average, 23 words were used by the respondents answering the questions. Content analysis led to the development of two categories embracing seven subcategories, with 26 codes being identified from the data (Table 1). To enhance the credibility of the analysis, quotations are presented in the text and the analytic track for each assigned category, subcategory and code is presented (Table 1).

### 3.1. Challenges in Applying the Regulations in Dog Trials and Competitions

This category comprises the four subcategories: 1. Tasks during trials and competitions; 2. The trial context; 3. Judges’ interpretations; and 4. Role of other officials. The respondents describe perceived elevated risks for injury, including challenging tasks, non-optimal surfaces, and ambiguities in the interpretation by judges and other officials.

#### 3.1.1. Tasks during Trials and Competitions

With regards to describing canine health and injury promotion, two points of view emerged. First, there were several tasks for the dogs to master that were commonly perceived and recurrently identified by some dog handlers as threats to canine health promotion and injury prevention. These included requirements for performance by the canine athlete, crawling, sitting and/or lying in a group with other dogs, protection work, high ladder, heavy retrieving, hurdle jump, and gun shots.

*“I feel the heavy dumbbells are too demanding for the dogs. In my case the dog is supposed to handle a 3 kg dumbbell while the dog itself only weighs 14 kg.”* (R715)

The opposite point of view noted was that other dog handlers expect to challenge the performance of their dog, highlighting potential long-term health benefits while rewarding optimal completion of the tasks.

*“The dogs’ performance should be tested. I don’t think it should be made easier but also not expose them to unnecessary stress.”* (R1284)

For instance, respondents expressed that by lowering demands of physical and mental capacity, e.g., by lowering obstacles and giving more time during tracking, an increased amount of dogs with poor structure and lower level of performance would be rewarded in competition. This would potentially lead to the selected breeding of dogs with lower inherited physical capacity and poor working ability.

*“Consider what simplifying the trial tasks can lead to in the long term, i.e., that if dogs are not able to handle being tested in physical and mental tasks to a sufficient degree it can over time lead to a ’weaker’ type of dog which will be more prone to injury and have less desire to work”* (R1101)

#### 3.1.2. The Trial Context

Respondents mentioned the environmental contexts of the tasks in the competition program, e.g., lack of time in the schedule as a potential barrier since they considered warm-up to be an essential part of the program during messenger and protection work trials. Lack of time between runs for messenger dogs was considered an important barrier due to poor recovery, an increased risk of injury in a state of fatigue, and a risk of mortality due to overheating in warm and humid climates. The respondents also expressed a need for guidance regarding appropriate running surfaces during agility and forest terrain, specifically during messenger and search and rescue trials.

*“Define hilly terrain more clearly. Some clubs interpret it as impassable terrain. This can lead to injury to the dog or handler.”* (R1252)

#### 3.1.3. Judges’ Interpretations

Many respondents expressed a desire for the judge to reward precision rather than speed in obedience tasks, such as in recall and retrieving of objects, in order to facilitate injury prevention. For several, consideration of the dogs’ individual body weight and height were requested prior to scoring. In some instances, the perceptions were that dogs’ ability to master tasks differs with regard to body structure and size when compared to other dogs. For example, the structure and conformation of a dog with a heavy body type makes it more physically challenging to master high velocity and high acceleration/deceleration compared to a dog with a lighter body type.

*“Lower the jumps for working dogs or demand better surfaces. That the judges really score each dog according to its physical abilities is very important. A heavy dog should not have to be trained to act as a lighter dog just to get a higher score. There is a big difference between a 37 kg male boxer and a 20 kg Border Collie. Working breeds must have a reasonable chance to be competitive if we want to continue seeing them in trials.”* (R1177)

#### 3.1.4. Role of Other Officials

Concerns were expressed that the strategy for safety in human-dog interaction by other officials might increase the risk of injury in dogs during protection work classes. The helpers’ approach to the dogs, their own safety and demonstrating an appropriate amount of threat rather than encouraging high speed was considered relevant.

*“More training and practice for Schutzhund decoys. Ideally less speed and more threat from the decoy to avoid collisions that are too powerful.”* (R601)

### 3.2. Implementation of Animal Welfare and Canine Well-Being Approaches

This category comprises three sub-categories: (1) dog handlers’ responsibility, (2) organizer’s integration, and (3) the lack of scientific research. Here the respondents describe the perceived importance of implementing animal welfare and canine well-being to dog sports and working dog trials and the need for scientific research.

#### 3.2.1. The Dog Handler’s Responsibility

There was a clear perception that the dog handler has the main responsibility for the well-being of a canine athlete and that the dogs should not be trialed on tasks that they are not properly prepared for.

*“The responsibility for the health and well-being of the dog must be with the owner and handler. We have to respect our teammates and best friends 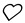
”* (R840)

Dog handlers’ knowledge, skills and attitudes were expressed as potential barriers as well as facilitators towards injury prevention.

*“You see too many times how the dog is taken directly from the car/resting and is immediately put to work without a warmup period or put back in the car after training, there is a lot of ignorance regarding health/training. I think it is the handler’s responsibility to keep the dog in good condition before competitions, training etc. I think the competitions are well designed (I can only speak of the venues I am participating in) as far as not causing injury to the dog."* (R1481)

Owner and handler education and knowledge of the most recent relevant research were expressed as keys to promoting health and well-being in sporting dogs.

*“I think it is important to be observant to discover injured dogs/dogs that are not 100% and that are entering [the trial or competition]. Difficult, each owner is responsible for the care of their dog the best way they know in order to not cause injury. Maybe classes/seminars about this specifically.”* (R1323)

Respondents suggested that improvements in breeding criteria and strategies would be the most effective facilitator of injury prevention.

*“This is a breeder question rather than a rulebook question. A body built for the power and a dog who engage the brain before acting is of good help.”* (R1351)

#### 3.2.2. Organizer’s Integration

Several participants suggested that there is a need to target canine working safety in humid and hot environments during trials and requested better access to hydration and cooling strategies.

*“To have additional recommendations for the responsible organizer regarding how to help dogs handle trials in different weather conditions, perhaps that trials can’t take place outdoors on icy grounds, and that water stations are available for field work etc.”* (R1464)

There was a concern that there is a spreading of bacterial and viral diseases between comingling dogs. Participants suggested and sought alternative methods of exhibiting retrieving tasks and preventing canine infectious disease, e.g., by avoiding shared retrieving objects between dogs.

*“Use individual dumbbells to reduce the spread of contagious diseases, for example kennel cough.”* (R1001)

Some respondents recalled having observed dogs with obesity or movement disorders participating in dog trials. Respondents encouraged organizers to disqualify dogs with apparent physical difficulties from the trials.

*“Encourage judges to excuse dogs who are not breathing properly”* (R1107)

Several expressed concern that the doping regulations were not respected and that dogs with orthopedic injury or disease, e.g., cranial cruciate ligament rupture, participate in trials and competitions without dispensation to compete post-surgically. Others suggested changes to the regulations, e.g., acceptance of using booties to protect paw pads and permission to remove dewclaws.

Pre-trial risk assessment of the terrain, surface and equipment were also requested. In particular, steep and impassable forest terrain was recalled and perceived as associated with an increased risk of injury in search and rescue and messenger dogs. Slippery surfaces, both indoors and outdoors, e.g., greens and steps at the high ladder, were also suggested as increasing the risk of falls and subsequent injuries.

*“No long down in groups with other dogs. I have gotten my second dog badly bitten by another dog in that exercise. Demand that the organizers not use slippery indoor flooring for the obedience exercises. I was recently forced to do just that at an obedience trial, the dogs had to run around a cone and take a jump on a slippery floor (just off the green mat that was on the premises).”* (R1369)

It was also suggested that judges and other officials be required to report unacceptable behavior in dogs and that greater consequences should be implemented for dogs with unacceptable behavior during trials.

*“Harder disciplinary actions against dogs that cause problems on the long down in the lower levels (novice). Higher demands on the judges and officials to report these dogs.”* (R1426)

There were comments that competitors should be encouraged by the organizer to warm-up their dogs prior to performing tasks and programs. They also expressed a need for a time schedule and a place to warm-up over the day of the trial.

#### 3.2.3. Lack of Scientific Research

Participants raised concerns regarding the lack of empirical evidence regarding risk factors for injury during training, sports- and working tasks. It was suggested that continuous risk evaluation should be performed with regards to the most recent scientific research during the recurrent revisions of utility and working dog trial regulations. Future studies addressing physical changes in breeding animals and performance was demanded.

*“Let knowledgeable veterinarians, and the statistics show what exercises cause most injuries. Change these exercises continually, as part of every revision of the rulebooks. The demands don’t need to be lowered, but the exercises should be adapted to fit dog ergonomics, to avoid future injuries.”* (R820)

## 4. Discussion

This qualitative research describes dog handlers’ perceptions regarding facilitators and barriers to health promotion and injury prevention in canine sports athletes participating in competitions and working trials. By allowing dog handlers to share their perceptions, we are able to demonstrate the value of experience in the field.

Acknowledging the extensive experience amongst canine sport handlers may increase the ability of the organizers of dog trials to identify and recognize important aspects of injury prevention and formulate effective negotiating strategies. According to respondents, there are a variety of aspects which can be perceived as facilitators and barriers of continuing health promotion related to canine working performance. Challenges in applying regulations in dog trials and competitions and lack of scientific research were barriers for health promotion and injury prevention. When considering specific tasks, there were two opposing perceptions regarding how to cope with the physical demands on the dogs. On one hand, respondents expressed that tasks might be made less physically demanding, e.g., by lowering obstacles, with reduced speed and more precision, reduced distance for the dog to crawl, and with lighter objects to retrieve. On the other hand, some respondents expressed that especially utility and working dogs must be confronted with reasonably challenging tasks for the breeds to maintain workability in the future. There are no empirical studies supporting or falsifying either of these perceptions [10,46].

However, there is a growing body of evidence within the field of canine sports and performance medicine. Bockstahler et al. [15] demonstrated that peak vertical force and vertical impulse increased in the forelimbs and decreased in the hind limbs in dogs retrieving objects at weights between 0.5 to 4 kg. Furthermore, the height of jumping obstacles has been found to affect joint angles and stress on the joints during landing [59,60]. Recent research on post-exercise cooling methods [25] and hydration strategies [26] have also been presented and require adherence by dog handlers and organizers of trials and competitions. Thus, it seems important to consider that canine performance at trial or competition relies not only on the dog’s physical and mental capacity. Furthermore, the dog trainer plays a major role in the outcome performance of sport and working dogs, since it is a consequence of the previous training strategies used.

The possibility to train their dogs ergonomically and with a reduced risk of injury or fear during working tasks may affect the respondents’ drive to exhibit and participate in working dog trials, e.g., search and rescue and protection work. Injuries or diseases occurring in canine sports athletes are significant negative consequences to dog handlers [61]. Past experience with harm associated with working tasks may limit the dog handlers’ perceived behavioral control of injury prevention. This may increase their intent to avoid injury in their dogs, but with activity and competition avoidance as a consequence [62,63].

Respondents expressed that dog handlers should not demand more from dogs than what they are prepared for, either physically or mentally. In serious leisure, self-determined extrinsic motivators for the dog handler, such as obtaining titles and/or rewards, may threaten dog welfare if the handler pursues performance without recognizing and respecting the insufficient or impaired capacity of the dog. Dog sport trials are serious leisure to some dog handlers and are mostly driven by intrinsic and self-determined extrinsic motivators [39,61,64,65]. Participation driven by extrinsic motivation is based on outcomes related to the activity, e.g., obtaining rewards, seeking approval and perceived value in participation. Intrinsic motivation-elicited participation in dog sports is based on the dog trainer’s desire to or interest in participating in an activity. Self-determined motivation is related to positive consequences such as enjoyment and pleasure. In addition, it is the dog handler’s responsibility to consult animal health care professionals if injury or disease is suspected in the dog. Based on the findings of this research, it is suggested that health assessments and treatment in canine sports and performance medicine should be conducted by appropriately educated animal health care professionals working with an evidence-based approach and under the Act on Professional Activities within the Field of Animal Health Care (2009:302) [66].

Furthermore, it has been observed in recent years that many welfare and wellness-focused products and activities are encouraged by social, commercial, or other entities which might financially benefit from such products and activities and which are not supported by scientific evidence [63,67]. The authors suggest caution to the dog owning and dog handling consumer with regard to use of these products and activities.

The dog handlers’ and the trial organizers’ recognition and integration of methods for responsible approaches to dog welfare were expressed by the respondents as facilitators to injury prevention and health promotion during SWDA working trials and competitions. One way to address this is to increase the competence of dog handlers regarding canine well-being and highlight the importance of form and structure in injury and disease prevention. Furthermore, breeding criteria can be improved and strategies to preserve physical capacity and performance in working dog breeds can be developed. All of this would apply to the Swedish Animal Welfare Act (Law 2018:1192) [43,44,55,67,68].

There is also an explicit responsibility with regard to animal welfare by the organizers of sport, utility and working dog trials, which is well-established in the Swedish Animal Welfare Act (Law 2018:1192) [55]. According to the Act, dogs should be protected from disease, and therefore the spreading of canine infectious disease, e.g., canine infectious respiratory disease [69]. This needs to be considered when dog trials and competitions are organized. In this study, respondents expressed that in tasks including the retrieval of objects, the object should be replaced with one that is clean. Hence, in order to reduce the transfer of bacterial and viral pathogens, the same object is not to be shared between dogs. General and specific regulations and guidelines developed by the FCI, kennel clubs and associated dog clubs need to be regularly and accordingly adjusted for the organizers to properly provide the competing dogs with a safe environment.

Among respondents in this study, there were several suggested actions to better implement animal welfare regulations. Suggestions regarding methods to reduce the risk of training and trialing dogs who might be subjected to suffering, i.e., showing signs of breathlessness, pain or anxiety, are in line with the Swedish Animal Welfare Act [55,70]. The availability of trial guidelines for working safely in hot and humid environments [24,25,26,71], pre-trial risk assessment of forest terrain, surface and equipment, and alteration of the schedule in order to facilitate injury preventive actions were requested. These are all actions which could be facilitated and encouraged by offering key guidelines to the local trial organizers. There was also the opinion that the organizers should practice their authority to disqualify dogs subjected to suffering during trial or competition. In line with excluding dogs from trials, the participants also expressed that doping and unacceptable behavior, e.g., aggression towards other dogs, needs to be controlled and that the consequences of such behaviors need to be reviewed.

There is a limitation to the interpretation of the results of this work. If interviews or focus groups were utilized rather than a survey, we might have gained more in-depth information from the respondents. Instead, we received a large number of responses, and data saturation was most certainly reached [72]. Even though the majority of respondents had previous experience from a dog being injured or diseased, there were still many respondents (30%) without this experience, which lead to a more heterogeneous sample and potentially a larger variability in the answers.

## 5. Conclusions

Canine health promotion and injury preventative priorities within the sport and working dog field were identified following a survey of dog handlers. Challenges in applying the regulations in dog trials and competitions and the lack of scientific research on specific tasks and normal physical changes and adaptation to stress were found to be barriers to injury prevention and health promotion in working and sport dogs. Animal welfare and canine well-being approaches implemented amongst stakeholders, i.e., the dog handlers and the organizer of competitions, were identified as facilitating factors. Issues relating to canine welfare and the special needs of sport and working dogs must be continuously addressed.

The findings in this study imply that the stakeholders must continuously strive to bridge possible gaps in the Swedish Animal Welfare Act based on scientific and empirical knowledge in canine sports and performance medicine. By refining the internal regulations and guidelines, and continuing to provide dog handler education, e.g., on breeding and various aspects of health and wellbeing, stakeholders may facilitate sustainability and soundness in sporting and working dogs. There is, however, a lack of epidemiological longitudinal studies and basic research targeting specific sport and working dog tasks.

## Figures and Tables

**Table 1:** Overview of categories, subcategories and codes merged from narrative data in the qualitative content analysis.

Category	Subcategory	Code
Challenges in applying the regulations in dog trials and competitions	Dog tasks during trials and competitions	Requirements in performance by the canine athlete
		Crawling
		Stay in a group with other dogs
		Protection work
		High ladder
		Heavy retrieving
		Jumping obstacles
		Gun shots
	The trial context	Time between runs for messenger dogs
		Need for definitions
	Judges’ interpretations	Rewarding precision rather than speed
		Considering individual characteristics
	Role of other officials	Interaction with the dog
		Safety strategy
Implemention of animal welfare and canine well-being approaches	Handler’s responsibility	Knowledge, skills and attitudes
		Breeding criteria and strategy
	Organizer’s integration	Working safely in hot and humid environment
		Prevention of the spreading of canine infectious disease
		Practice authority to discontinue a dog during trial and competition
		Doping in dog sports and training
		Reporting unacceptable behavior
		Pre-trial risk assessment of the forest terrain and surface
		Facilitation of health promoting actions/interventions
		Safe equipment
	Lack of scientific research	Documented risk during specific tasks
		Normal physical changes in dogs

## Data Availability

The data presented in this study are available on request from the corresponding author.
